# Nocturnally migrating songbirds drift when they can and compensate when they must

**DOI:** 10.1038/srep21249

**Published:** 2016-02-16

**Authors:** Kyle G. Horton, Benjamin M. Van Doren, Phillip M. Stepanian, Wesley M. Hochachka, Andrew Farnsworth, Jeffrey F. Kelly

**Affiliations:** 1 Department of Biology, University of Oklahoma, Norman, Oklahoma, USA; 2 Oklahoma Biological Survey, University of Oklahoma, Norman, Oklahoma, USA; 3 Advanced Radar Research Center, University of Oklahoma, Norman, Oklahoma, USA; 4 Department of Ecology and Evolutionary Biology, Cornell University, Ithaca, New York, USA; 5 School of Meteorology, University of Oklahoma, Norman, Oklahoma, USA; 6 Cornell Lab of Ornithology, Ithaca, New York, USA

## Abstract

The shortest possible migratory route for birds is not always the best route to travel. Substantial research effort has established that birds in captivity are capable of orienting toward the direction of an intended goal, but efforts to examine how free-living birds use navigational information under conditions that potentially make direct flight toward that goal inefficient have been limited in spatiotemporal scales and in the number of individuals observed because of logistical and technological limitations. Using novel and recently developed techniques for analysis of Doppler polarimetric weather surveillance radar data, we examined two impediments for nocturnally migrating songbirds in eastern North America following shortest-distance routes: crosswinds and oceans. We found that migrants in flight often drifted sideways on crosswinds, but most strongly compensated for drift when near the Atlantic coast. Coastal migrants’ tendency to compensate for wind drift also increased through the night, while no strong temporal differences were observed at inland sites. Such behaviors suggest that birds migrate in an adaptive way to conserve energy by assessing while airborne the degree to which they must compensate for wind drift.

How do birds migrate in unfavorable winds? Although migration has fascinated scientists for millennia^1^,^2^,^3^,^4^,^5^,^6^,^7^,^8^, this fundamental question about the behavior of billions of migrating birds remains unresolved. Decades of research have yielded contradictory results on how migrants cope with adverse wind conditions, whether they use common strategies in such situations, and how important these behaviors are to an organism’s fitness^2^,^3^,^4^,^5^,^9^,^10^. Recent studies have demonstrated that migrants can choose when to fly to avoid adverse conditions and enhance travel speeds^11^,^12^,^13^. Their successful arrival at breeding or wintering grounds depends on the capacity of migrants to make time-sensitive decisions on how to orient to exploit wind patterns in order to maximize energetic efficiency and minimize lateral drift^10^,^11^.

Birds can avoid drifting off course by preferentially migrating in favorable tailwind conditions^14^,^15^,^16^,^17^,^18^; however costs (both time and energetic) may be incurred if tailwinds are infrequent^18^,^19^,^20^. Alternatively, birds can initiate flight under wind regimes with crosswind components at the cost of being drifted away from optimal north-south migration routes. In-flight migrants can use one of two strategies in crosswinds: they can maintain a constant heading towards their destination and allow crosswinds to influence their resultant flight tracks (Fig. 1a); or they can counter a crosswind by orienting (i.e., heading) in an offset position, a strategy known as compensation (Fig. 1b). Although compensation minimizes overall flight distance, diminished groundspeeds that result from flying in crosswinds may actually render this a suboptimal strategy^21^. Conversely, fully drifting birds can use their full heading vector to maximize groundspeed, at the cost of geographic displacement, which can reduce overall migration speed, increase energetic expenditure, and result in decreased fitness^3^,^12^,^22^,^23^.

Despite potential advantages for detours and variation in migration timing^24^,^25^, encounters with inhospitable terrains (e.g. deserts, large lakes, seas, oceans) may account for significant mortality^26^,^27^,^28^. Furthermore, longer duration flights that result from drift may take migrants further from key stopover habitats and delay arrival on breeding grounds, and both of these errors may be costly at the individual level^24^. Over small spatial scales (e.g. using single radars), birds have been shown to partially compensate for wind drift in unfavorable winds^18^,^22^ and to exhibit within-night shifts in the mean track of nocturnal migration preceding a water crossing^29^,^30^,^31^, suggesting an active shift in migrant motivation. However, the means to test hypotheses regarding these flight strategies, particularly at coherent regional and full-nightly scales, have not existed until recently. In particular, diagnosis of a migrant’s heading (body axis direction)−key to understanding flight strategies−was until recently only possible using vector subtraction of estimated wind from measured track.

The upgrade of the United States national weather radar network to dual-polarizations is allowing direct determination of migrant heading (body axis direction) and track (the resultant direction of bird movement given wind motion) to assess long-standing theoretical predictions of these behaviors^32^,^33^. Here, using recently developed techniques for analysis of Doppler polarimetric weather radar data, we test the prediction that nocturnal migrant songbirds compensate for wind drift and that this compensation will be more extreme near an ocean barrier than over a contiguous continental land mass.

## Results and Discussion

We examined strategies of nocturnally migrating birds using Doppler polarimetric radars at three coastal and three inland sites in the eastern United States during autumn of 2013 and 2014 (Fig. 2). Each radar site provides independent scans of migrants’ headings and tracks for areas nearing 49,000 km^2^. Radars collected data every five to ten minutes, yielding approximately 1.6 million samples from 55 nights (Supplementary Table S1).

The typical directions of headings and tracks of birds was toward the southwest (Fig. 3a). Tracks were more southerly than birds’ headings, indicating that on average birds were being drifted by crosswinds. The difference between heading and track was 33.66° at inland sites and 42.32° near the coast; the smaller difference at inland sites indicates a greater propensity of birds to drift sideways (Fig. 3a). We found that birds flying near the Atlantic coast increasingly oriented and tracked westward, away from the coast, with each subsequent decile of the night (direction of heading 2.24° per decile more westward, and direction of travel 2.37° per decile; Fig. 3a). In contrast, birds flying over inland sites showed near-zero changes in both the headings and directions they flew with each subsequent decile of the night (direction of heading –0.03° per decile, and direction of travel 0.06° per decile; Fig. 3a).

Migrants at inland sites displayed moderate to high propensity to drift (slope of alpha: 0.63–0.77, Fig. 3b), whereas migrants at coastal sites showed both an overall lower propensity to drift (slope of alpha: 0.29–0.65, Fig. 3b) and a change in the magnitude of drift through the night. At coastal sites, the propensity to drift decreased through the night, and behaviors diverged markedly after the middle of the night (i.e. decile 5, Fig. 3b). Migrants preferred direction of movement (PDM) showed little variability across the night at inland sites (mean ± 95% CI 206.41 ± 8.27 to 212.02 ± 5.56°) in comparison to a 2.32° per decile increase in PDM at coastal sites (mean ± 95% CI 209.22 ± 6.32° to 232.68 ± 8.11°).

Typical nocturnal winds blew to the southeast, and southwest-bound birds consistently oriented across these winds to the west and partially compensated for coastward wind drift. In conditions of prevailing crosswinds, a partial compensation strategy can increase ground speeds to expend less energy per unit distance^3^,^22^. When winds were east of the PDM, migrant heading and track differed significantly (paired test of means, coastal & inland: *P *< 0.0001; Fig. 4a,b), whereas differences were not evident when winds were west of the PDM (paired test of means, coastal: *P *= 0.14, inland: *P *= 0.69; Fig. 4c,d).

The prediction that migrants compensate more for drift when encountering a migration barrier is consistent with these results. Birds over inland sites without ecological barriers compensated on average for only 29.0% of the effect of wind, whereas birds near coastal sites compensated for drift to an increasingly greater extent over the course of the night, reaching the highest average level of wind drift compensation (74.5%) during decile 10 (Fig. 3a). Aversion to a water crossing close to sunrise and into the daylight hours may be a product of dwindling fat stores through the night and atmospheric changes after sunrise that make migration less efficient for most birds^21^,^34^. Previous research with orientation cages, individual releases, and radio tracking has established that birds with substantive fat stores are likely to orient in directions that would bring them over a barrier, whereas those lacking sufficient fat usually avoid such barriers^35^,^36^,^37^,^38^,^39^. Over smaller spatial scales, within-night shifts in the mean track of nocturnal migration precede a water crossing^29^,^30^ and active inland reorientation occurs near coasts^40^,^41^,^42^. However, no studies have captured the large-scale phenomena we documented using weather radars. Analyses at this scale are based on detection of upwards of 5 million migrating birds (mean ± 95% CI 1,034,440 ± 42,668; Supplementary Table S3), thus representing the behavioral response of a significant fraction of the migrant bird assemblage.

Whether birds migrate when winds are unfavorable and to what degree they compensate for resulting drift have been long-standing questions in migration biology^2^,^3^,^4^,^5^,^6^,^12^. We show for the first time at a regional scale, in a regularly and heavily traveled airspace of the Nearctic-Neotropic migration system, that birds routinely migrate under crosswind conditions and compensate in a context specific manner. This result is consistent with migrants knowing their location relative to migration barriers while in flight and actively assessing the degree to which they need to compensate for wind. Since Doppler radar averages headings and tracks within radial bands, the extent to which birds uniformly or heterogeneously re-orient to original or updated preferred directions is not clear. These changes in behavior may be facilitated by visual cues (e.g. rivers and coasts)^43^,^44^, compass direction^45^,^46^, and likely the interaction of multiple sensory systems. Regardless of the biological cues used for active assessments, our results strongly suggest that migrants choose to drift, not compensate, under a wide range of winds when they face no impending inhospitable barrier.

New independent measures of migrant heading provided by polarimetric data significantly improve our ability to quantify migrant behavior at regional to continental scales. Increasing automation of radar analysis will further enable exploration and quantification of the full complement of United States weather radar data to achieve real-time monitoring of the phenology, distribution, abundance, and behaviors of billions of birds during their biannual migrations. Although greatly underused, the U.S. weather surveillance radar network provides the largest sensor array worldwide for monitoring animal migration (i.e. birds as well as bats and insects). These analyses will fill gaps in our understanding of migratory behaviors at large scales while fulfilling a primary requirement to shed light on past, present, and future behavioral strategies of aerial taxa.

## Methods

### Weather surveillance radar data

We examined migrant flight patterns at six weather surveillance radars (WSR-88D): three coastal and three inland sites (Fig. 2). The radars transmit at a wavelength of 10 cm (S-band), peak power of 750 kW, and sample (i.e. scan) 360° every 5 to 10 minutes depending on the volume coverage pattern (VCP). The VCP specifies the operational elevation angles of the antenna (e.g., 0.5°, 1.5°, …, 19.5°) and the temporal update time. Radars sampled the airspace at range intervals of 250 m at 0.5° azimuthal intervals (720 radials) from 2–230 km in range. We acquired 2013 and 2014 Level-II data products from August 1^st^ to November 15^th^ from the National Centers for Environmental Information (NCEI) archive (http://www.ncdc.noaa.gov/has/has.dsselect). We visually screened data from all nights to discard scans with weather contamination and anomalous propagation and restricted analyses to samples for the period between evening and morning civil twilight (sun angle 6° below horizon)^47^. We aggregated all measures (track, heading, migration intensity, and bird abundance) to tenths of the night (hereafter “deciles”). In addition to data quality measures described below, we included only nights containing measures from at least four radars. After screening and data quality protocols we retained 55 of 214 potential sampling nights from August 6^th^ to October 30^th^.

### Track

We generated velocity azimuth displays (VAD) from ~ 0.5° elevation angle radial velocity measures to estimate ground speed and track direction of flying animals. We followed Sheldon *et al*.^48^ to dealias velocities when necessary and Browning and Wexler^49^ to estimate ground speed and track direction for each range annulus. Radial velocities required dealiasing when the inbound or outbound speeds of targets exceeded the Nyquist velocity of the radar^48^. We included estimates up to 2 km above ground level (a.g.l.; ~125 km range), retaining only those estimates with root mean squared error less than 5. We aggregated height profiles of flight track by column averaging. We estimated target airspeed by:
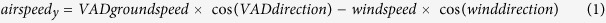

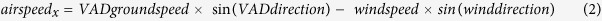

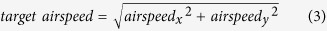
Nightly airspeeds across radar stations averaged 7.83 m/s, and pooled nightly mean airspeeds were greater than 4.5 m/s.

### Heading

**Figure 1:**
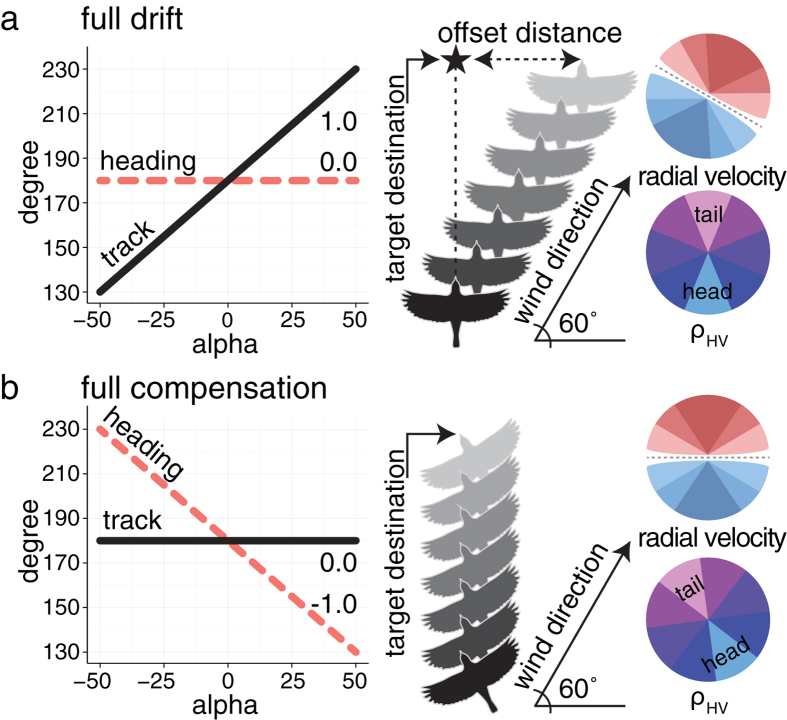
Conceptual models for migrant flight behaviors. Generalized statistical (left), flight (middle), and radar (right) interpretations of (**a**) full drift and (**b**) full compensation. Full drift is characterized by a slope of 1 when monitoring track in relation to alpha and 0 when monitoring heading. Drift signifies a change in track with changing wind parameters but no change in migrant heading. For this reason, flight track is directed towards the prevailing wind direction. For simplicity, bird airspeeds are ignored. Track measures represented by radial velocity, blue (negative) indicating approaching targets and red (positive) representing targets receding from the radar. Radar correlation coefficient (ρHV) differentiates migrant head and tail features to measure heading.

**Figure 2:**
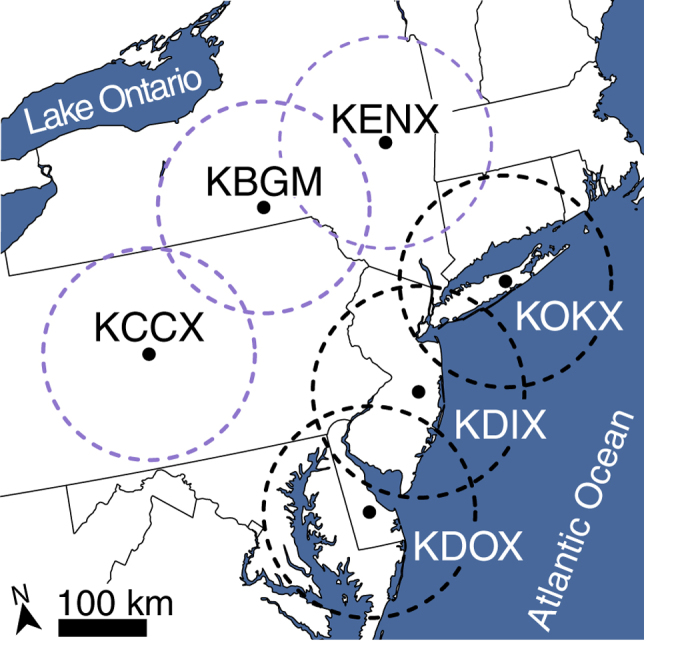
Radar sampling regions. Radar locations and biological ranges (125 km) denoted by circles. Purple rings indicate inland classification and black coastal. Autumn data from 2013 and 2014 were assessed from August 6^th^ to October 30^th^. Map was generated using QGIS^57^.

**Figure 3:**
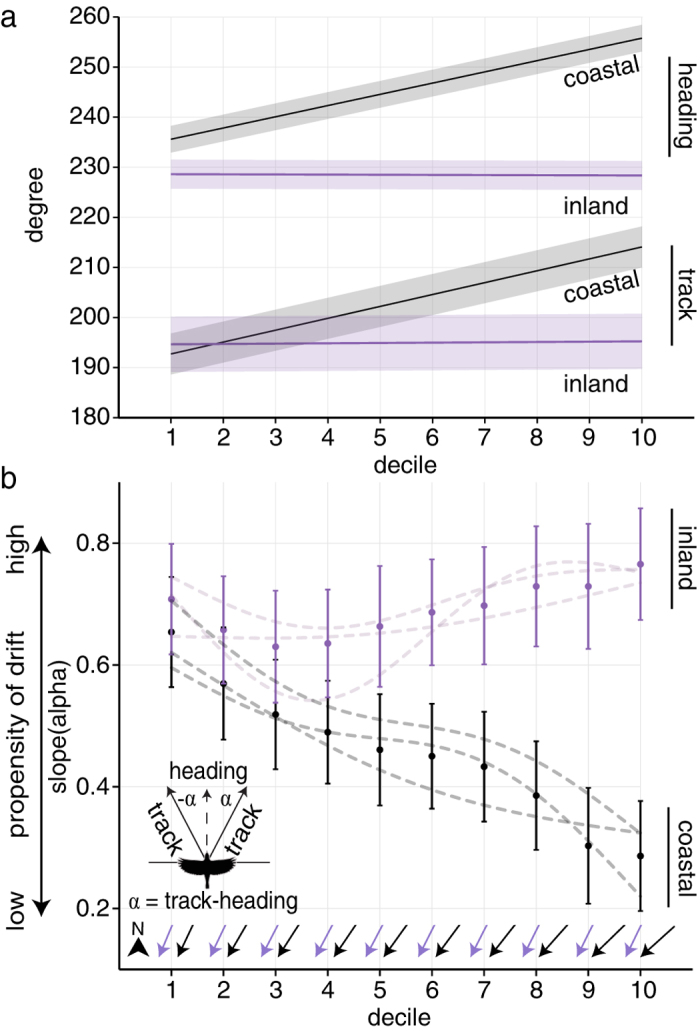
Migrant flight behaviors. (**a**) Modeled mean heading and track directions as inferred by GAMM to account for fixed and random spatiotemporal effects. Birds followed mean tracks between 203.56–204.91° at coastal sites and 190.07–203.64° at inland sites (Supplementary Table S2). Birds’ headings were further west than they traveled, between 241.60–252.06° for coastal sites and 226.26–229.71° for inland sites (Supplementary Table S2). We found differences in means of coastal and inland track directions (LMM: *P *= 0.038) as well as heading directions (LMM: *P *< 0.001). Linear change in migrant heading and track for coastal and inland regions revealed significant temporal shifts in coastal track (GAMM: *P *< 0.001) and heading (GAMM: *P *< 0.001). Inland sites showed non-significant, near-zero changes in track (GAMM: *P *= 0.763) and heading (GAMM: *P *= 0.804). Wind heading was a significant non-parametric factor for all cases (GAMM: *P *< 0.01). (**b**) Mixed-effect model output depicting migrant behavior through the night for coastal and inland regions. Higher values of the slope of alpha indicate a stronger propensity for a drift behavior (0 = full compensation; 1 = full drift). Transparent lines represent site-specific behaviors and error bars 95% confidence intervals. Arrows represent preferred direction of movement. Individual radar coefficients interpolated using a generalized additive model.

We determined migrant heading using the co-polar cross-correlation coefficient (ρ_HV_) radar product from the ~0.5° tilt angle scans following Stepanian and Horton^33^ (Fig. 1). We fit models to three sequential range gates (250 m intervals from the radar − 750 m in total) across all azimuths to ensure sufficient data for extraction. We eliminated individual volumes, the smallest sampling unit for WSR-88Ds, with non-biological characteristics (i.e. –33 dBZ) and estimated heading only for ranges with more than 300 azimuthal samples. We visually inspected all heading extractions to 125 km in range (2 km a.g.l.) to ensure that automation captured well-defined symmetry axes. We then projected range measures (125 km) to height above ground level at 10 m intervals. This process yielded multiple extractions (three to five) per height interval. At each interval we included heading extractions that explained, on average, greater than 15% of the variance^33^ (when fitting ρ_HV_ to a sinusoid) and an average standard deviation in heading direction that was less than 20°. These criteria typically removed scans with light migratory movements, movements in which birds may have oriented in many different directions (i.e., low directional alignment), and those in close proximity to weather systems.

### Relative migration intensity and abundance

To assess relative nightly migration intensity we calculated average reflectivity factor (dBZ) from the ~0.5° tilt angle from 5–150 km from each radar. To reduce underestimates of migration intensity, we omitted all clear-air echo returns (–33 dBZ) in our averaging process. We weighted all statistical analyses by migration intensity.

To estimate migrant abundance, we derived the number of birds for each ~0.5° tilt angle sweep from 20 to 125 km following Chilson *et al*.^50^. To mitigate clutter contamination we used more distant starting range gates and omitted volumes with greater than 35 dBZ. Reflectivity factor (dBZ) was converted to dBη following: η[dB] = Z[dBZ] + β (4), where β = 10log_10_(10^3^π^5^|K_m_|^2^/λ^4^) (5). We used an average WSR-88D wavelength (λ) of 10.7 cm and |K_m_|^2^ for liquid water of 0.93, the dielectric constant. This yielded β = 13.37. We chose a cross section (σ) of 17.5 cm^2^, representative of songbirds^51^, to convert η to birds/km^3^. To extract the number of birds per sweep we calculated the volume of each range gate as follows:
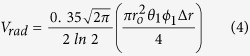
where 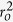 is the mid-range of the range gate, Δ*r* equals the range gate spacing (250 m), and 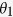 and 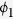 the half power beam width (0.96°). We aggregated measures of bird abundance to nightly averages.

### Quantifying wind speed and direction

**Figure 4:**
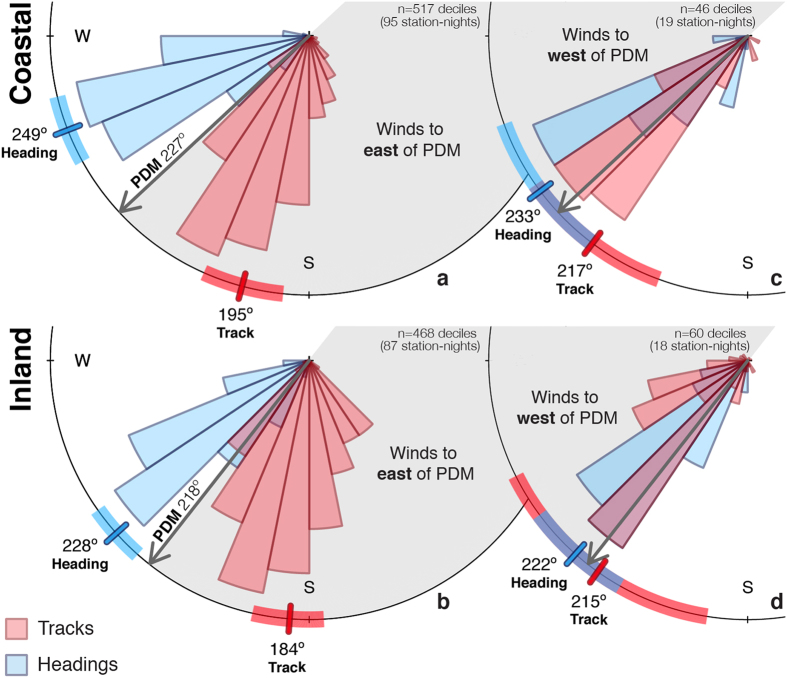
Heading and track distributions. Migrating birds’ tracks and headings for winds east (**a**,**b**) and west (**c**,**d**) of the preferred direction of movement (PDM). The area of each sector is proportional to the frequency of directions in that sector, weighted by migration intensity (dBZ). Mean directions plotted as tick marks on the circle border, 95% confidence intervals shown as transparent rectangles behind tick marks. Mean heading and track directions were calculated from decile samples.

We gathered nightly pressure level gridded North American Regional Reanalysis (NARR, http://wwwt.emc.ncep.noaa.gov/mmb/rreanl/index.html) pressure and monolevel data to estimate winds aloft within the radar coverage areas^52^. Wind speed and direction are mapped at a 32 km spatial resolution and updated every three hours. We used pressure level measures to calculate speed and direction of winds aloft from *u* (zonal velocity; east-west) and *v* (meridional velocity; north-south) measures from 2 isobaric levels: 900 and 950 hPa. We used monolevel surface geopotential height data to determine site-specific ground-level pressure levels. We linked all radar measures with the closest temporal wind measurements. Because coastal and inland sites differed in height above sea level (mean height above sea level ± SD; inland: 593.0 ± 125.8 m; coastal: 28.3 ± 15.3 m), we used 950 hPa winds (mean height ± 95% CI, 573.14 ± 2.34 m a.g.l.) for coastal sites and 900 hPa for inland sites (mean height ± 95% CI, 630.77 ± 3.57 m a.g.l.). For analyses of wind scenarios east and west of the PDM (Fig. 4), only winds with speeds greater than 5 m/s were included because they yielded consistent (low standard deviation) wind directions within the sampling region.

### Statistics

We conducted statistical analyses in R, version 3.0.2^53^, with GAMM implemented using the mgcv package^54^ and linear mixed models implemented using the lme4 package^55^.

### Generalized additive mixed model (GAMM)

To examine the temporal variation of migrant heading and track, we used a generalized additive mixed model. Because migrant behavior tends to covary with winds aloft, we used a non-parametric spline fit for wind direction, and decile as a fixed effect. We used a single random effect of the interaction of year, radar station, and ordinal date.

### Linear mixed models (LMM)

Alpha, the difference between a bird’s track and its heading, provides information about the extent to which birds compensate for wind drift^32^. This relationship defines migrants’ preferred direction of movement (PDM)^6^,^56^ and measures migrant flight strategy via the slope of alpha (0 = complete compensation, 1 = complete drift; Fig. 1a,b). Intermediate values represent a mixture of these behaviors (i.e. partial compensation for drift). Our two fixed effects addressed the temporal and site-specific features of drift propensity: 1) region (coastal or inland) and 2) the interaction of alpha, region, and decile. We used multiple levels of random effects to account for non-independence among samples. We included three random slope and intercept terms: 1) interaction of year, radar station, and ordinal date, 2) interaction between year and radar station, and 3) ordinal date. In addition to accounting for pseudoreplication from temporally correlated samples, this random effect structure statistically incorporated variation in drift propensity and PDM over time and space, leaving the fixed effects to describe the average patterns in which we were interested. We used 2000 bootstrapped replicates to estimate 95% confidence intervals.

We implemented a similar mixed model approach to test for mean differences in heading and track across coastal and inland regions, modeling heading or track as a function of region. We included random intercepts following the same structure as above with the addition of decile as a random effect. To calculate means of migrant heading and track, we used mixed models, accounting for non-independence of samples by designating random effects of decile and sampling period for each station.

## Additional Information

**How to cite this article**: Horton, K. G. *et al*. Nocturnally migrating songbirds drift when they can and compensate when they must. *Sci. Rep.* **6**, 21249; doi: 10.1038/srep21249 (2016).

## Supplementary Material

Supplementary Information

## References

[b1] Aristotle. History of animals: Books VII–X. Translated by D. M. Balme. Cambridge, Mass: Harvard University Press. (1991).

[b2] Evans P. R. Migration and orientation of passerine night migrants in northeast England. J. Zool. 150, 319–348 (1966).

[b3] Alerstam T. & Hedenström A. The development of bird migration theory. J. Avian Biol. 29, 343–369 (1998).

[b4] Thorup K., Alerstam T., Hake M. & Kjellen N. Bird orientation: compensation for wind drift in migrating raptors is age dependent. Proc. R. Soc. B 270, S8–11 (2003). 10.1098/rsbl.2003.0014 PMC1698035 12952622

[b5] Gauthreaux S. A. & Able K. P. Wind and the direction of nocturnal songbird migration. Nature 228, 476–477 (1970). 16058549 10.1038/228476a0

[b6] Chapman J. W. . Animal orientation strategies for movement in flows. Curr. Biol. 21, R861–R870 (2011). 22032194 10.1016/j.cub.2011.08.014

[b7] Chapman J. W. . Flight orientation behaviors promote optimal migration trajectories in high-flying insects. Science 327, 682–685 (2010). 20133570 10.1126/science.1182990

[b8] Alerstam T. & Petterson S. Do birds use waves for orientation when migrating across the sea? Nature 259, 205–207 (1976).

[b9] Sergio F. . Individual improvements and selective mortality shape lifelong migratory performance. Nature 515, 410–413 (2014). 25252973 10.1038/nature13696

[b10] Liechti F. Birds: blowin’ by the wind? J. Ornithol. 147, 202–211 (2006).

[b11] McLaren J. D., Shamoun-Baranes J., Dokter A. M., Klaassen R. H. G. & Bouten W. Optimal orientation in flows: providing a benchmark for animal movement strategies. J. R. Soc. Interface. 11, 20140588 (2014). 25056213 10.1098/rsif.2014.0588 PMC4233736

[b12] Chapman J. W. . Adaptive strategies in nocturnally migrating insects and songbirds: contrasting responses to wind. J. Anim. Ecol. n/a–n/a (2015).doi: 10.1111/1365-2656.12420 26147535

[b13] Chapman J. W. . Detection of flow direction in high-flying insect and songbird migrants. Curr. Biol. 25, R751–R752 (2015). 26325133 10.1016/j.cub.2015.07.074

[b14] Able K. P. The flight behaviour of individual passerine nocturnal migrants: a tracking radar study. Anim. Behav. 25, 924–935 (1977).

[b15] Larkin R. P. & Thompson D. Flight speeds of birds observed with radar: Evidence for two phases of migratory flight. Behav. Ecol. Sociobiol. 7, 301–317 (1980).

[b16] Erni B., Liechti F., Underhill L. G. & Bruderer B. Wind and rain govern the intensity of nocturnanl bird migration in central Europe — a log-linear regression analysis. Ardea 90, 155–166 (2002).

[b17] Schaub M., Liechti F. & Jenni L. Departure of migrating European robins, *Erithacus rubercula*, from a stopover site in relation to wind and rain. Anim. Behav. 67, 229–237 (2004).

[b18] Alerstam T. Optimal bird migration revisited. J. Ornithol. 152, 5–23 (2011).

[b19] Wikelski M. . Avian metabolism: Costs of migration in free-flying songbirds. Nature 423, 704–704 (2003). 12802324 10.1038/423704a

[b20] Thorup K., Alerstam T., Hake M. & Kjellén N. Traveling or stopping of migrating birds in relation to wind: an illustration for the osprey. Behav. Ecol. 17, 497–502 (2006).

[b21] Alerstam T. Wind as selective agent in bird migration. Ornis Scand . 10, 76–93 (1979).

[b22] McLaren J. D., Shamoun-Baranes J. & Bouten W. Wind selectivity and partial compensation for wind drift among nocturnally migrating passerines. Behav. Ecol. 23, 1089–1101 (2012). 22936843 10.1093/beheco/ars078 PMC3431116

[b23] Kranstauber B., Weinzierl R., Wikelski M. & Safi K. Global aerial flyways allow efficient travelling. Ecol. Lett. 18, 1338–1345 (2015). 26477348 10.1111/ele.12528

[b24] Hahn S. . Variable detours in long-distance migration across ecological barriers and their relation to habitat availability at ground. Ecol Evol 4, 4150–4160 (2014). 25505540 10.1002/ece3.1279 PMC4242566

[b25] Arlt D., Olsson P., Fox J. W., Low M. & Pärt T. Prolonged stopover duration characterises migration strategy and constraints of a long-distance migrant songbird. Animal Migration 2, 47–62 (2015).

[b26] Schmaljohann H., Liechti F. & Bruderer B. Songbird migration across the Sahara: the non-stop hypothesis rejected! Proc. R. Soc. B 274, 735–739 (2007). 10.1098/rspb.2006.0011 PMC2197203 17254999

[b27] Diehl R. H., Bates J. M., Willard D. E. & Gnoske T. P. Bird mortality during nocturnal migration over Lake Michigan: a case study. Wilson J. Ornithol. 126, 19–29 (2014).

[b28] Lok T., Overdijk O. & Piersma T. The cost of migration: spoonbills suffer higher mortality during trans-Saharan spring migrations only. Biol. Lett. 11, 20140944 (2015). 25589489 10.1098/rsbl.2014.0944 PMC4321157

[b29] Zehnder S., Åkesson S., Liechti F. & Bruderer B. Nocturnal autumn bird migration at Falsterbo, south Sweden. J. Avian Biol. 32, 239–248 (2001).

[b30] Fortin D., Liechti F. & Bruderer B. Variation in the nocturnal flight behaviour of migratory birds along the northwest coast of the Mediterranean Sea. Ibis 141, 480–488 (1999).

[b31] Peterson A. C., Niemi G. J. & Johnson D. H. Patterns in diurnal airspace use by migratory landbirds along an ecological barrier. Ecol. Appl. 25, 673–684 (2014). 26214913 10.1890/14-0277.1

[b32] Green M. & Alerstam T. The problem of estimating wind drift in migrating birds. J. Theor. Biol. 218, 485–496 (2002). 12384051

[b33] Stepanian P. M. & Horton K. G. Extracting migrant flight orientation profiles using polarimetric radar. IEEE Trans. Geosci. Remote Sens. 53, 6518–6528 (2015).

[b34] Richardson W. J. Wind and orientation of migrating birds: a review. Experientia 60, 226–249 (1991). 10.1007/978-3-0348-7208-9_11 1838517

[b35] Backman J., Pettersson J. & Sandberg R. The influence of fat stores on magnetic orientation in day-migrating chaffinch, *Fringilla coelebs*. Ethology 103, 247–256 (1997).

[b36] Deutschlander M. E. & Muheim R. Fuel reserves affect migratory orientation of thrushes and sparrows both before and after crossing an ecological barrier near their breeding grounds. J. Avian Biol. 40, 85–89 (2009).

[b37] Sandberg R. Interaction of body condition and magnetic orientation in autumn migrating robins, *Erithacus rubercula*. Anim. Behav. 47, 679–686 (1994).

[b38] Sandberg R., Moore F. R., Bäckman J. & Lõhmus M. Orientation of nocturnally migrating Swainson’s thrush at dawn and dusk: importance of energetic condition and geomagnetic cues. Auk 119, 201–209 (2002).

[b39] Deppe J. L. . Fat, weather, and date affect migratory songbirds’ departure decisions, routes, and time it takes to cross the Gulf of Mexico. PNAS 201503381 (2015). doi: 10.1073/pnas.1503381112 PMC4655507 26578793

[b40] Able K. P. The orientation of passerine nocturnal migrants following offshore drift. Auk 94, 320–330 (1975).

[b41] Richardson W. J. Northeastward reverse migration of birds over Nova Scotia, Canada, in autumn: a radar study. Behav. Ecol. Sociobiol. 10, 193–206 (1982).

[b42] Bruderer B. & Liechti F. Flight behavior of nocturnally migrating birds in coastal areas — crossing or coasting. J. Avian Biol. 499–507 (1998).

[b43] Bingman V. P., Able K. P. & Kerlinger P. Wind drift, compensation, and the use of landmarks by nocturnal bird migrants. Anim. Behav. 30, 49–53 (1982).

[b44] Cochran W. W. & Kjos C. G. Wind drift and migration of thrushes: a telemetry study. Illinois Natural History Survey Bulletin 33, 297–330 (1985).

[b45] Able K. P. & Able M. A. Development of sunset orientation in a migratory bird: no calibration by the magnetic field. Anim. Behav. 53, 363–368 (1997).

[b46] Deutschlander M. E. & Muheim R. In Encyclopedia of Animal Behavior (ed. Moore M. D. B. ) 314–323 (Academic Press, 2010). at < http://www.sciencedirect.com/science/article/pii/B9780080453378003636>

[b47] Farnsworth A. . A characterization of autumn nocturnal migration detected by weather surveillance radars in the northeastern US. Ecol. Appl. (2015). doi: 10.1890/15-0023.1 27411248

[b48] Sheldon D. . Approximate bayesian inference for reconstructing velocities of migrating birds from *weather radar*. AAAI 1334–1340 (2013).

[b49] Browning K. A. & Wexler R. The determination of kinematic properties of a wind field using Doppler radar. J. Appl. Meteor. 7, 105–113 (1968).

[b50] Chilson P. B. . Estimating animal densities in the aerosphere using weather radar: To Z or not to Z? Ecosphere 3, art72 (2012).

[b51] Larkin R. P. Flight speeds observed with radar, a correction: slow ‘birds’ are insects. Behav. Ecol. Sociobiol. 29, 221–224 (1991).

[b52] Mesinger F. . North American Regional Reanalysis. Bull. Amer. Meteor. Soc. 87, 343–360 (2006).

[b53] R Core Team. R: a language and environment for statistical computing. R Foundation for Statistical Computing. (2014). at <Available at: http://www.r-project.org>.

[b54] Wood S. *mgcv: Mixed GAM Computation Vehicle with GCV/AIC/REML Smoothness Estimation*. (2015). at 〈http://cran.r-project.org/web/packages/mgcv/index.html〉.

[b55] Bates D., Mächler M., Bolker B. & Walker S. Fitting Linear Mixed-Effects Models using lme4. *arXiv:1406.5823 [stat]* (2014). 〈http://arxiv.org/abs/1406.5823〉.

[b56] Kemp M. U. . Quantifying flow-assistance and implications for movement research. J. Theor. Biol. 308, 56–67 (2012). 22683380 10.1016/j.jtbi.2012.05.026

[b57] QGIS Development Team. QGIS Geographic Information System. (Open Source Geospatial Foundation, 2015). at < http://qgis.osgeo.org>.

