# Supplementary material for: Nocturnally migrating songbirds drift when they can and compensate when they must
Source: Sci Rep. 2016 Feb 16;6:21249. doi: 10.1038/srep21249 (PMC4754638; doi:10.1038/srep21249)
Supplement: Supplementary Information [file srep21249-s1.doc]

Title: Nocturnally migrating songbirds drift when they can and compensate when they must

**Authors:** Kyle G. Horton^1,2,3^*, Benjamin M. Van Doren^4^, Phillip M. Stepanian^3,5^, Wesley M. Hochachka^6^, Andrew Farnsworth^6^, and Jeffrey F. Kelly^1,2^

**Supplementary Tables:**

**Supplementary Table S1. Sample sizes of radar measures of heading and track**. Samples sizes collected for heading and track measures. Measures are collected every 250 m in range from the radar. All preserved measures met screening criteria described in methods.

| *Inland* | 2013 | 2014 | Total |
| --- | --- | --- | --- |
| Radar | *n* | *n* | *n* |
| KBGM;  Binghamton, NY | 167,121 | 184,030 | 351,151 |
| KENX;  Albany, NY | 82,444 | 115,203 | 197,647 |
| KCCX;  State College, PA | 144,738 | 97,269 | 242,007 |
| *Coastal* | 2013 | 2014 | Total |
| Radar | *n* | *n* | *n* |
| KDIX;  Mt. Holly, NJ | 152,909 | 149,308 | 302,217 |
| KDOX;  Dover, DE | 143,844 | 137,493 | 281,337 |
| KOKX;  New York, NY | 103,270 | 120,421 | 223,691 |
| Total | 794,326 | 803,724 | 1,598,050 |

**Supplementary Table S2. Mean fall heading and track directions.** Heading and track directions for Inland and coastal radar sites weighted by migration intensity (dBZ). Bootstrapped 95% confidence intervals in parentheses.

| *Inland* | Mean Heading (degree)  (95% CI) | Mean Track (degree)  (95% CI) |
| --- | --- | --- |
| KBGM;  Binghamton, NY | 229.71°  (224.85, 234.60) | 190.07°  (182.29, 198.45) |
| KENX;  Albany, NY | 226.26°  (219.63, 232.46) | 192.25°  (183.49, 200.84) |
| KCCX;  State College, PA | 227.60°  (221.63, 233.73) | 203.64°  (195.38, 212.92) |
| *Coastal* | Mean Heading (degree)  (95% CI) | Mean Track (degree)  (95% CI) |
| KDIX;  Mt. Holly, NJ | 244.53°  (238.96, 250.41) | 204.91°  (196.47, 213.98) |
| KDOX;  Dover, DE | 241.60°  (234.64, 248.63) | 203.59°  (195.85, 210.84) |
| KOKX;  New York, NY | 252.06°  (244.55, 260.02) | 203.56°  (195.01, 212.27) |
|  |  |  |

**Supplementary Table S3. Migrant abundance within sampling regions.** Mean and range of migrant birds within the sampling region of each radar site (20-125 km). Means and ranges based on nightly averages. Number of birds calculated using a cross-section of 17.5cm^2^, representative of songbirds^51^. 95% confidence intervals in parentheses.

| *Inland* | 2013 | | | 2014 | | |
| --- | --- | --- | --- | --- | --- | --- |
| Radar | Mean birds  (±95% CI) | Range of  birds | Sampling  nights | Mean birds  (±95% CI) | Range of  birds | Sampling  nights |
| KBGM;  Binghamton, NY | 601,784  (±146,773) | 54,553 to  5,245,856 | 22 | 447,842  (±64,057) | 55,312 to  2,291,963 | 28 |
| KENX;  Albany, NY | 872,263  (±159,171) | 169,560 to  5,467,045 | 18 | 614,674  (±86,336) | 36,042 to  2,630,611 | 23 |
| KCCX;  State College, PA | 716,796  (±134,147) | 69,071 to  5,491,108 | 10 | 348,084  (±56,628) | 42,810 to  1,736,133 | 22 |
| *Coastal* | 2013 | | | 2014 | | |
| Radar | Mean birds  (±95% CI) | Range of  birds | Sampling  nights | Mean birds  (±95% CI) | Range of  birds | Sampling  nights |
| KDIX;  Mt. Holly, NJ | 1,606,117  (±169,060) | 225,224 to  5,209,128 | 23 | 1,011,452  (±188,222) | 50,270 to  5,477,626 | 30 |
| KDOX;  Dover, DE | 528,723  (±82,920) | 87,230 to  2,924,113 | 20 | 500,336  (±121,354) | 61,712 to  3,669,654 | 29 |
| KOKX;  New York, NY | 920,513  (±117,751) | 240,696 to  3,898,720 | 22 | 937,613  (±188,392) | 24,266 to  5,238,237 | 28 |
